# p53 pathway dysfunction in AML: beyond *TP53* mutations

**DOI:** 10.18632/oncotarget.22713

**Published:** 2017-11-27

**Authors:** Sean M. Post, Steven M. Kornblau, Alfonso Quintás-Cardama

**Affiliations:** Alfonso Quintás-Cardama: TCR2 Therapeutics, Cambridge, MA, USA

**Keywords:** TP53, Mdm2

In recent years, a multitude of studies have shed light regarding the genomic, transcriptomic, and epigenetic architecture of multiple human cancers including myeloid malignancies such as acute myeloid leukemia (AML). Yet, comparatively little is known regarding the (phospho)proteomic abnormalities carried by AML cells that are responsible for the phenotypic features of the disease and play a critical role in prognosis and resistance to therapy. Deletions and/or mutations at the *TP53* locus can be detected in approximately 5% of patients with newly diagnosed AML [1]. Such genomic alterations represent one of the most powerful independent prognostic factors in AML [2], which highlights the key role played by p53 dysfunction in the pathogenesis of that malignancy. Of note, *TP53* mutations are present in AML subclones at diagnosis and become dominant upon relapse post chemotherapy. This occurrence indicates that standard cytotoxic therapy does not induce *TP53* mutations. Rather, it selects for mutated clones that are chemotherapy-resistant and expand preferentially during therapy [3].

Quintás-Cardama et al have recently reported that, in addition to somatic mutations, p53 dysfunction can arise via aberrant expression of proteins that regulate p53 stability and function (e.g. overexpression of its canonical negative regulators Mdm2 and/or Mdm4) [4]. The authors’ central hypothesis was that wild-type p53 function must be compromised in a high proportion of patients carrying wild-type *TP53* alleles. In order to demonstrate the latter, they used a proteomics platform to systematically characterize variations in protein expression of different elements in the p53 pathway in a large cohort of patients with newly diagnosed AML. That systems biology approach coupled with computational analysis of interactomes and mutational profiling allowed the authors to draw novel conclusions with critical clinical implications. First, while p53 stabilization was unsurprisingly found to be a universal phenomenon among mutant *TP53* samples, the former was also frequently observed in wild-type *TP53* samples, and more importantly, it predicted for an equally dismal prognosis regardless of *TP53* mutational status. Second, Mdm2 was frequently overexpressed in AML cells bearing wild-type *TP53* alleles, and results in as dismal a prognosis as that reported for patients whose AML cells exhibit p53 stabilization, be it through *TP53* mutations or otherwise. In that regard, Mdm2 overexpression could be considered a functional surrogate of p53 protein dysfunction. Finally, AML samples harbor unique patterns of p53 pathway protein expression, which allows for segregation into prognostic groups with distinct long-term remission and cure rates. The latter is important because different prognostic groups are linked to different patterns of protein expression both within as well as outside of the p53 pathway. When examined in detail, such distinct patterns of protein activation contain potential AML vulnerabilities that can be therapeutically exploited.

To explore this further, Quintás-Cardama *et al* demonstrated that reactivation of p53 functions via Mdm2-antagonists in the context of Mdm2 overexpression and wild-type p53 restored p53’s anti-tumor effects. This finding may be relevant beyond AML for the clinical management of other malignancies where p53 functions have been shown to be attenuated in the absence of *TP53* point mutations or 17p deletions. For instance, *TP53* mutations are typically present in only 5-10% of *de novo* B- and T-cell acute lymphoblastic leukemia (B- and T-ALL) [5]. However, alterations in critical regulators of the p53 pathway are extremely common. In Philadelphia positive (Ph+) B-ALL, deletion of the *CDKN2A* locus (harboring the *p14*^*ARF*^ gene -a critical negative regulator of MDM2) has been observed in 40% of *de novo* cases [6]. This genetic loss represents a powerful mechanism to ablate the anti-tumor functions of wild-type p53 and suggests the patients with Ph+ ALL that harbor haploinsufficient or homozygous *CDKN2A* loss may be amenable to therapeutic strategies identified by Quintás-Cardama *et al.* Furthermore, and in addition to hematologic malignancies, concomitant overexpression of Mdm2 and/or Mdm4 in the context of wild-type p53 has been observed in nearly 50% of patients with head and neck squamous carcinomas [7]. These results are strikingly similar to the observations made by Quintás-Cardama et al and further support their notion that therapeutic modalities that “re-engage” wild-type p53 functions may be therapeutically exploited to improve clinical outcomes.

The findings reported by Quintás-Cardama et al are particularly relevant because they are translatable into clinical practice. Patients with AML carrying *TP53* mutations are at the highest risk of death and, if eligible, they are customarily recommended to undergo allogeneic stem cell transplantation or participation in a clinical trial. If prospectively confirmed, Quintás-Cardama’s findings suggest that such recommendation could be applied to patients with AML exhibiting p53 protein stabilization and to those with Mdm2 protein overexpression. In the latter case, a recommendation to enroll in trials testing experimental agents should include those with Mdm2 blocking activity.
